# Joint trajectories of life style indicators and their links to psychopathological outcomes in the adolescence

**DOI:** 10.1186/s12888-021-03403-y

**Published:** 2021-08-17

**Authors:** Anhui Zhang, Jiao Fang, Yuhui Wan, Puyu Su, Fangbiao Tao, Ying Sun

**Affiliations:** 1 Department of Child Health Care, Wuhu Maternal and Child Health (MCH) Center, Wuhu, 230000 Anhui Province China; 2 Department of Maternal, Child & Adolescent Health, School of Public Health, Anhui Medical University, Hefei, 230032 Anhui Province China; 3 MOE Key Laboratory of Population Health Across Life Cycle, Hefei, 230032 Anhui Province China

**Keywords:** Lifestyle pattern, Longitudinal trajectory, Psychopathology, Pubertal transition

## Abstract

**Background:**

Rapid socio-economic development makes China a unique laboratory for examining how lifestyle changes affect adolescent mental health. This study aims to identify joint trajectories of modifiable lifestyle indicators during pubertal transition and its associations with psychopathological outcomes.

**Methods:**

A cohort of 1974 children aged 7–9 years were recruited in Anhui Province, China during March 2013. The assessment of lifestyle behaviors (screen time, physical activity, sleep duration and beverage intake) and depressive symptoms were conducted from Wave 1 to Wave 4 (2018). Suicide ideation, non-suicidal self-harm (NSSI) and alcohol use were self-reported at Wave 4. Longitudinal trajectories of lifestyle patterns were defined using group-based multi-trajectory models in 2019.

**Results:**

Four lifestyle trajectories were identified: persistent healthy (39.9%), suboptimal healthy (25.3%), unhealthy mitigation (17.2%), and persistent unhealthy (17.7%). Compared with persistent healthy group, the risk of subsequent suicide ideation [odds ratio (OR): 2.86, 95%CI: 2.15–3.81], depressive symptoms (OR: 2.16, 95%CI: 1.39–3.35), alcohol use (OR: 2.53, 95%CI: 1.78–3.61) and non-suicidal self-harm (OR: 1.35, 95%CI: 1.09–1.67) was significantly higher in persistent unhealthy group.

**Conclusions:**

This study provided convincing evidence that unhealthy lifestyle trajectory during adolescence is associated with more than two-fold elevated odds for multiple domains of psychopathological outcomes over 5 years.

**Supplementary Information:**

The online version contains supplementary material available at 10.1186/s12888-021-03403-y.

## Background

The past three decades brought tremendous changing in the socio-economic context of adolescent development in China with implications for mental health. China is heading towards a ballooning epidemic of mental health problems in teens [1, 2]. The nationwide psychiatric survey among 73,992 participants aged 6–16 years of age in China suggests that the weighted prevalence of any disorder was 17.5% [3]. The 12-month prevalence of non-suicidal self-injury (NSSI), suicidal ideation and suicide attempt was 26.1, 17.5 and 4.4%, respectively, based on a population of 14,820 students aged 10–20 years from three provinces in China [1].

Such soaring of mental health problem has paralleled a dramatic shift in health-related behaviors and lifestyle from traditional, healthy patterns towards unhealthy patterns. During the transition from childhood to adolescence, children in China gradually develop its unique lifestyle pattern due to high level academic pressure compared with their counterparts in western countries, including prolonged sitting and screen time [4, 5], high proportion of regular sugar-sweetened beverages (SSBs) consumption [6] and insufficient sleep [7].

The role of lifestyle in the causation of mental health has long been established and provoked recommendations regarding diet, sedentary activity (primarily screen time), physical activity (PA), and sleep [8]. Recent meta-analyses of prospective studies also concluded inverse associations of PA with depression in youth [9] and children [10]. Our previous work explored the potential importance of eating patterns for mental health in 14,500 adolescents at grade 7–12 from 32 schools in 4 provinces across China [11], and found a significant dose-response relationship between SSBs consumption pattern with higher risk of psychological symptoms during adolescence. A recent population-based cohort study in Norway, Trondheim Early Secure Study, reported an association between short sleep duration and increased risk of future incidence of emotional disorder symptoms in both boys and girls and between reduced sleep and behavioral disorder symptoms in boys [12].

Previous studies have demonstrated that adolescents are more likely to engage in a spectrum of lifestyle risk behaviours, such as high sugar-sweetened beveages consumption, being physically inactive, engaging in sedentary behaviour and excessive screen time, which may have synergistic effects on health [13, 14]. The majority of studies focused on individual behavior [15] or summed multiple behaviors into an index score, e. g. a lifestyle risk index [7].

Adolescents establish patterns of behavior and make lifestyle choices that affect both their current and future health and well-being [16, 17]. The onset of adolescence represents an important turning point in an individual’s life, not only from a psychological point of view, but also with regard to the emergence of psychopathology [18]. Given the importance of this transitional period and the need for targeted preventive efforts, as well as the high prevalence of depressive symptoms, non-suicidal self-injury and suicide behaviors among Chinese children and adolescents [1, 3], the aim of this longitudinal study was to gather information on developmental trajectory of a combination of modifiable, health-related behaviors among Chinese children over the transition to adolescence, and to evaluate their associations with self-destructive behaviors (non-suicidal self-harm, suicide ideation) and depressive symptoms.

## Methods

### Study design and participants

**Fig. 1:**
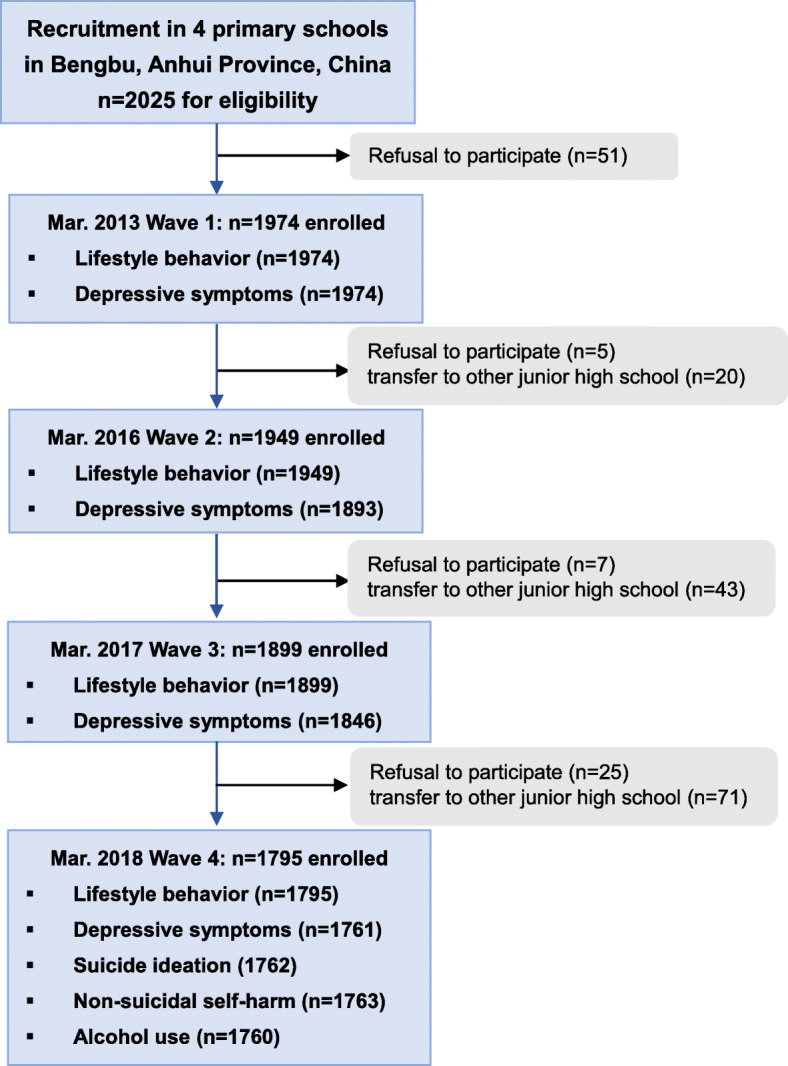
Flowchart of Participants Follow-up

The present study used data of an ongoing longitudinal study examining psychosocial determinants of growth and development in Anhui Province, China. As illustrated in Fig. 1, of a total of 2025 students in grade 1 to 3 from four large elementary schools of Bengbu city, 1974 (1104 boys, 55.9%) agreed to participate. Bengbu city, located in north Anhui Province of China. The gross domestic product per capita (GDP) increased by over 12% annually, doubling the figure of the former period before 2010. It is one of the representative cities in China in terms of its urbanization and economic development.

Baseline survey started in March 2013, and subsequent waves of data collection took place 2 years (wave 2), 4 years (wave 3) and 5 years (wave 4) later. We secured approval from Institutional Review Boards at Anhui Medical University and then obtained written informed consent from parents and school teachers, as well as child assent. Parents of the students completed baseline questionnaire survey at home. In subsequent waves, questionnaires were completed by students in class at school with supervising research assistants available to answer questions following a standard script.

This study was approved by the Medical Ethics Committee of Anhui Medical University. All the study participants and their guardians provided written informed consent. All the methods in this study were in accordance with the institutional research committee and tenets of the Declaration of Helsinki.

### Measurements

#### Lifestyle factors

##### Physical activity

Youth physical activity levels were ascertained from the Youth Risk Behavior Survey 2013 (YRBS) at each wave [19]. Parents (wave 1) and adolescents (wave 2-wave 4) were asked “During the past 7 days, on how many days were your child (you) physically active for a total of at least 60 minutes per day? (Add up all the time you spent in any kind of physical activity that increased your heart rate and made you breathe hard some of the time.)”.

##### Screen time

Weekly average screen time was derived based on daily time (hours) spent on screen during weekdays and weekends (including watching television/video, playing computer, iPad and smartphone), which was calculated as: weekly average screen time = (weekday screen time × 5 + weekend screen time × 2)/7.

##### Sugar-sweetened beverage consumption (SSBs)

Sugar-sweetened beverage consumption was assessed though the question ‘During the past 7 days, how many times did your child (you) drink at least 1 serving regular sugar-sweetened sodas, fruit drink, sweetened iced tea, sports /energy drink that contains sugar?’

##### Sleep duration

Sleep duration was estimated by subtracting self-reported waketime from bedtime, assessed by asking, “On a usual weekday this past week, when did your child (you) go to bed at night?” and “On a usual weekday this past week, when did your child (you) wake up the next morning?” Average sleep duration was calculated as: average sleep duration = (weekday sleep duration × 5 + weekend sleep duration × 2)/7.

#### Outcomes

##### Baseline depressive symptoms assessment

The Short Mood and Feelings Questionnaire Parent-report (SMFQ-P) has been widely used in children aged 6 to 17 years, including our previous work [20–22]. Cronbach’s alphas for our sample were high (0.85). Scores on SMFQ-P range from 0 to 26, we defined depressive symptoms as having an SMFQ-P score of 11 or greater [22].

##### Follow-up depressive symptoms assessment

Self-reported depressive symptoms over the past 2 weeks were assessed by means of the Mood and Feelings Questionnaire (MFQ) at wave 2–4 [23]. The MFQ has shown prognostic validity in clinic and non-clinic samples [24], yielding high internal consistency (α = 0.91–0.93) in the present sample. MFQ scores range from 0 to 66, adolescents with total scores of 29 or above was considered depressive [25]. The split-half reliability was 0.90, the Cronbach α was 0.93, and the test-retest reliability was 0.84 in Chinese children and adolescents [26].

##### Suicide ideation

At wave 4 (2018), a single-item question was used to assess suicide ideation (“Have you ever seriously thought about killing yourself and, if so, have you had these thoughts in the past 12 months?”) from the World Mental Health CIDI (WMH-CIDI) [27].

##### Non-suicidal self-harm behaviors

At wave 4 (2018), adolescents were asked “Have you ever harmed yourself in a way that was deliberate, but not intended as a means to take your life in the last (reference period)?” [28]. A list of eight non-suicidal self-harm (NSSI) (hitting, pulling hair, banging head, pinching, scratching, biting, firing/burning, cutting) methods was then presented. The internal consistency reliability of NSSI was 0.780 in Chinese adolescents [29].

##### Alcohol use

Alcohol use at wave 4 (2018) was defined as having at least one drink of beer, wine, or liquor (not just a sip or a taste of someone else’s drink) during the past 30 days [30].

#### Covariates

##### Body mass index

At each wave, height and weight was measured and body mass index (BMI) was calculated as weight (in kg) divided by height squared (in m).

##### Parental education and family monthly income

Parents at baseline reported educational attainment during the consent process and family monthly income from “1” for “< 2000 RMB” (ca. 313 US$) to “5” for “> 15,000 RMB” (ca. 2345 US$).

##### Warm parenting

All parents were questioned a 13-item scale adapted by Raudino et al. [31] from the Child Rearing Practices Report and the Parenting Scale. Cronbach’s alpha in the current study was 0.91. The warm parenting score ranges from 0 ~ 52, with higher scores indicating a more supportive and nurturing parental relationship (parental warmth).

##### Adverse childhood experiences

At wave 4 (2018), adolescents reported experiences with adverse events including abuse and neglect by using the 10-item Adverse Childhood Experiences Questionnaire - Short Form (ACES-SF) [32]. Eight items from this measure were included. A total score with higher values indicating greater experience of childhood adversity. Cronbach’s alpha in the current study was 0.75.

### Statistical analysis

#### Descriptive

The study sample was characterized using descriptive statistics and frequency distributions at baseline and across following three waves. Average level of four lifestyle behaviors over time were analyzed by three-way repeated measures analysis of variance (ANOVA) with time, sex, family income, maternal education, and weight status as factors. Multiple comparisons were assessed by Bonferroni post-hoc test.

#### GBTM model selection

Group-based multi-trajectory modeling, a generalization of univariate group-based trajectory modeling to multiple outcomes [33], was adopted to examine latent clusters of children with similar lifestyle trajectories across the four lifestyle behaviors: weekly screen time, physical activity, sleep duration, and SSBs consumption. For counting variables of ‘physical activity for 1h/day’ and ‘SSBs consumption’, the zero-inflated Poisson (ZIP) model was used. For continuous variables of ‘weekley screen time’ and ‘sleep duration’, cencered normal model was used. The number of classes that best fit was selected based were identified based on the acceptability of the available fit-criteria indices (i.e., Bayesian Information Criterion [BIC], sample-size-adjusted BIC [SSABIC], Akaike Information Criterion, Supplementary Table 1), model parsimony, distinctiveness of temporal patterns via visual inspection and cluster size.

#### Associations between multiple trajectories and psychopathological outcomes

After the lifestyle multi-trajectory profiles were identified, preliminary analyses calculated descriptive statistics and tested lifestyle trajectory group differences in terms of depressive symptoms across four waves, as well as suicide ideation, alcohol use and non-suicidal self-harm assessed in Wave 4. We then examined associations between longitudinal changes in depressive symptoms and lifestyle trajectory groups using mixed-effects logistic growth modeling, controlled for time, age, sex, BMI, family monthly income, low maternal education, warm parenting score and childhood adverse experiences (these predictor variables are uncorrelated with each other based on collinearity analysis). Multiple logistic regression model was performed to examine association between suicide ideation, alcohol use and non-suicidal self-harm with four lifestyle trajectory groups. Linearity in the multivariable models were checked upon for continuous predictors. In these models, the four lifestyle trajectories were treated as nominal variables with the persistent healthy lifestyle class serving as the reference group. Analyses were performed using STATA Software Version 14 (College Station, TX: StataCorp LP; 2015). A *P*-value < 0.05 was considered statistically significant.

## Results

### General information

Table 1 describes baseline characteristics of the 1974 children (mean age 8.1 years, SD 0.87 at baseline) were included. More than half (55.9%) were male. Over one-fourth of children at baseline were overweight (16.2%) or obese (12.4%). Less than 5% of the participants come from low-income families. Those who did not provide information on depressive symptoms, suicide ideation, NSSI and alcohol use had higher BMI and were older compared to those followed up. No significant differences were found in proportion of boys, baseline SMFQ score and family monthly income between missing and follow-up groups, except that boys were more likely have missing data on depressive symptoms in wave 2 (Appendix Table 2).

**Table 1:** Descriptive Analysis of Lifestyle Measures across Four Waves among Chinese Children, Mean (SD)

	Wave 1	Wave 2	Wave 3	Wave 4
Participants, nm	1974	1949	1899	1795
Boys	1098 (55.6)	1078 (55.3)	1060 (55.8)	1001 (55.8)
Family monthly income				
< 2000 RMB	80 (4.1)	79 (4.1)	76 (4.0)	74 (4.1)
2000–5000 RMB	1061 (53.7)	1053 (54.0)	1021 (53.8)	960 (53.5)
5000–10,000 RMB	633 (32.1)	621 (32.0)	611 (32.2)	581 (32.4)
10,000–15,000 RMB	151 (7.6)	148 (7.6)	145 (7.6)	138 (7.7)
> 15,000 RMB	49 (2.5)	48 (2.5)	46 (2.4)	42 (2.3)
Maternal education				
< high school	463 (23.5)	459 (23.6)	437 (23.0)	405 (22.6)
high school	972 (49.2)	957 (49.1)	937 (49.3)	900 (50.1)
bachelors/ graduate	539 (27.3)	533 (27.3)	525 (27.6)	490 (27.3)
Body mass index, kg/m^2^	16.75 ± 2.9	19.79 ± 3.7	20.26 ± 3.9	20.93 ± 4.0.
ST, h/d	2.94 (1.8)	2.71 (2.1) ^§^	2.17 (1.9) ^§^	1.92 (1.4) ^§^
PA ≥1 h/d, d/w	2.38 (1.9)	2.63 (2.0) ^‡^	3.60 (1.8) ^§^	3.52 (1.7) ^§^
Sleep duration, h	9.39 (0.5)	9.16 (0.6) ^§^	8.53 (0.7) ^§^	8.06 (0.7) ^§^
SSBs≥1 serving, d/w	1.51 (2.0)	1.78 (1.8) ^§^	2.55 (1.9) ^§^	**–**

*Abbreviations*: *ST* screen time, *PA* physical activity, *SSBs* sugar-sweetened beverages

† *P* < 0.05; ‡ *P* < 0.01; § *P* < 0.001

### Group based multi-trajectory analysis of lifestyle patterns

**Fig. 2:**
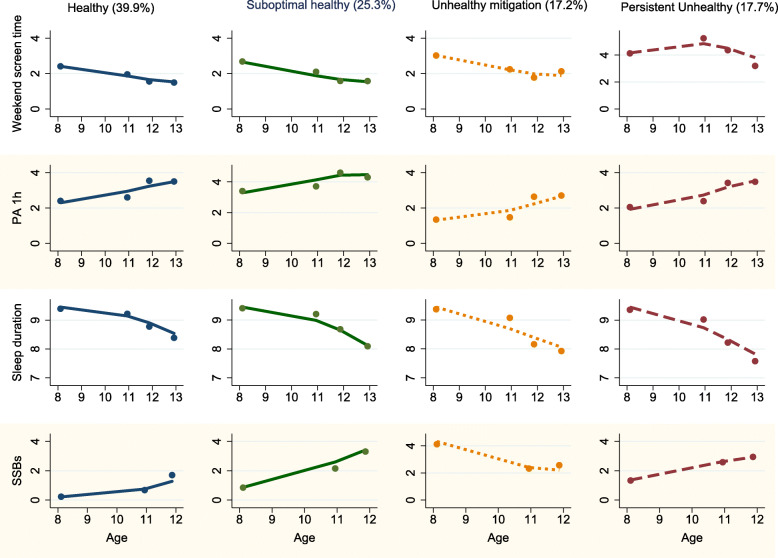
Multi-trajectory analysis of lifestyle behaviors among Chinese adolescents in a 5-year longitudinal study

Multi-trajectory analysis of the longitudinal behavior data on 1974 children revealed four distinct trajectories of lifestyle patterns (model fit statistics see **Appendix Table** **1**). Figure 2 showed the estimated mean levels in the four health-related behaviors at four waves for each trajectory group.

About 39.9% (802/1974) of children maintained a stable-low level of screen time (2.4 h/d to 1.4 h/d, in wave 1 and wave 4, respectively; the same hereinafter) and SSBs intake (0.2 times/wk. to 1.7 times/wk) with high-increasing physical activity (2.4 d/wk. to 3.5 d/wk), albeit the general decreasing trend in sleep duration (9.4 h/d to 8.4 h/d), throughout the 5-year follow-up (persistent healthy group).

More than one in four children (439/1974, 25.3%) were classified as suboptimal healthy group who started with healthy behaviors, but then experienced a substantial increase in SSBs intake (0.8 times /wk. to 3.3 times/wk) and a slightly shorter sleep duration (9.4 h/d to 8.1 h/d);

Nearly 17.2% (332/1974) of children were classified as “unhealthy mitigation group”, characterized with high-decreasing screen time (3.0 h/d to 2.1 h/d), low-increasing physical activity (1.3 d/wk. to 2.6 d/wk) and high-decreasing SSBs intake (4.4 times/wk. to 2.5 times /wk), albeit decreasing trend in sleep duration (9.4 h/d to 8.0 h/d);

About 17.7% (401/1974) of children were identified as “persistent unhealthy group”, characterized with excessive screen time (4.2 h/d to 3.2 h/d), abrupt decrease in sleep duration (9.4 h/d to 7.5 h/d) and stable-high SSBs intake (1.3 times/wk. to 3.2 times/wk), albeit increasing physical activity (2.0 d/wk. to 3.4 d/wk). The mean posterior probability for each trajectory group exceeded 0.92, while other fit statistics also confirmed that the four latent trajectory groups were optimal for our study data.

### Association of four lifestyle trajectory groups with psychopathological outcomes

Table 2 described the comparison of prevalence in depressive symptoms, suicide ideation, alcohol use and NSSI across trajectory groups. The prevalence of depressive symptoms across four waves was significantly higher in the unhealthy persistent group compared to both the persistent healthy and the suboptimal healthy groups (6.7% vs. 2.5 and 1.6% at baseline, *P* < 0.05; 21.3% vs. 11.3 and 8.3% at wave 4, *P* < 0.001).

**Table 2:** Prevalence of depressive symptoms, suicide ideation, alcohol use and non-suicidal self-harm across four lifestyle trajectory groups in the longitudinal lifestyle data on 1974 children

Outcomes	Participants	Prevalence n (%)	Lifestyle Multi-trajectories ^**a**^ [N (%)]			
			Healthy	Suboptimal Healthy	Unhealthy Mitigation	Unhealthy Persistent
**General**	1974		787 (39.9)	499 (25.3)	339 (17.2)	349 (17.7)
**Low maternal education**^**b**^	1974		156 (19.5)	95 (21.6)	93 (27.9) ^§^	119 (29.7) ^§^
**Low household income**^**c**^	1974		26 (3.4)	11 (3.5)	17 (4.1)	22 (5.6)
**More than 1 ACE**			206 (28.8)	130 (32.8)	115 (39.4) ^‡^	148 (41.3) ^§^
**Warm parenting score (0–15)**			11.3 ± 3.7	11.3 ± 3.8	10.5 ± 4.1 ^‡^	10.1 ± 4.3 ^§^
**Depressive symptoms**						
Baseline	1974	75 (3.8)	20 (2.5)	7 (1.6)	21 (6.3) †	27 (6.7) †
Wave 2	1893	116 (6.1)	29 (3.8)	13 (3.1)	17 (5.4)	57 (14.5) §
Wave 3	1846	184 (10.0)	53 (7.0)	21 (5.1)	38 (12.5) †	72 (19.4) §
Wave 4	1761	230 (13.1)	67 (9.3)	32 (8.1)	55 (18.8) §	86 (21.3) §
**Suicide ideation**	1762	191 (10.8)	50 (7.1)	39 (8.6)	41 (13.8) §	61 (19.9) §
**Alcohol use**	1760	105 (6.0)	25 (3.5)	25 (5.5)	19 (6.4) †	36 (11.8) §
**Non-suicidal self-harm**	1763	403 (22.9)	138 (19.5)	103 (22.7)	68 (22.8)	94 (30.7) ‡
**Missing**						
Wave 2	1749	25 (1.3)	12 (1.5)	7 (1.4)	4 (1.2)	2 (0.6)
Wave 3	1899	75 (4.0)	31 (4.1)	7 (1.2)	21 (6.2)	16 (4.6)
Wave 4	1795	179 (9.1)	73 (9.3)	30 (6.0)	37 (10.9)	39 (11.2)

^a^Compared with healthy lifestyle persistent group, † *P* < 0.05; ‡ *P* < 0.01; § *P* < 0.001

^b^Equal or lower than junior middle school;

^c^Household income equal or lower than 2000 yuan per month

Approximately 1 in 5 (19.9%) children reported suicide ideation in the unhealthy persistent group, compared with 1 in 14 (7.1%) in healthy group (*P* < 0.001). Alcohol use and NSSI were more frequent in the unhealthy persistent compared to the healthy groups (11.8% vs. 3.5% for alcohol use, *P* < 0.001; 30.7% vs. 19.5% for NSSI, *P* < 0.01).

A similar pattern was observed in the unhealthy mitigation group (Table 2). Depressive symptoms, suicide ideation and alcohol use over 5 years follow-up were significant higher compared with healthy groups (18.8% vs. 9.3% for depressive symptoms, *P* < 0.001; 13.8% vs. 7.1% for suicide ideation, *P* < 0.001; and 6.4% vs. 3.5% for alcohol use, *P* < 0.01).

Similar attrition rates were observed across healthy, mitigation and persistent unhealthy lifestyle trajectories (Table 2).

### Prediction of psychopathological outcomes by the four distinct trajectory groups

**Fig. 3:**
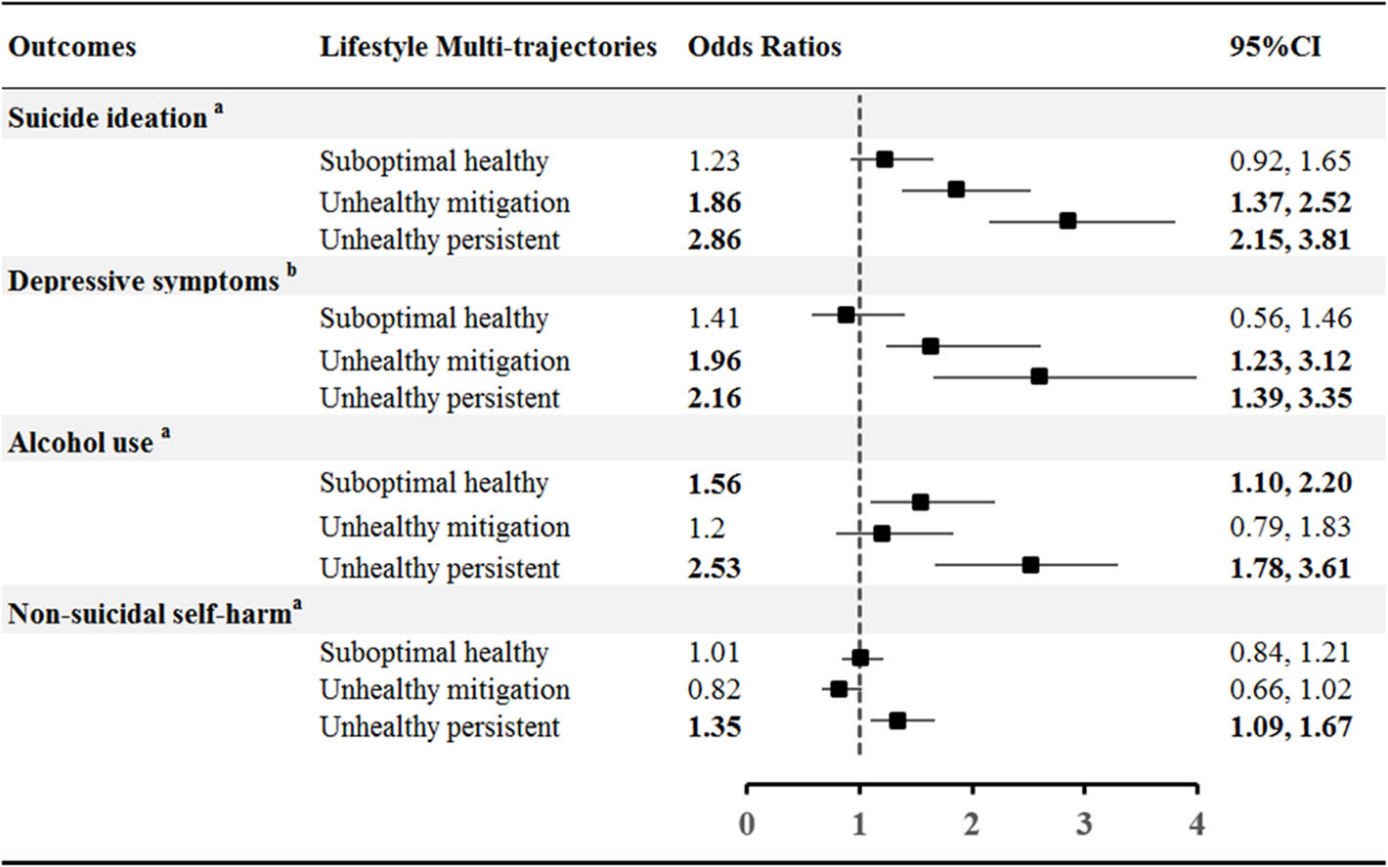
Adjusted odds ratios with 95% confidence intervals for suicide ideation, depressive symptoms, alcohol use and non-suicidal self-harm in different lifestyle trajectory groups. Bold numbers indicate significant differences when using the healthy group as the reference category; ^a^ adjusted for depressive symptoms, age, BMI, sex, household income, maternal education, warm parenting and adverse childhood experiences in Wave 4, by using logistic regression models. ^b^ adjusted for fixed effects of age, BMI, sex, household income, maternal education, warm parenting, and adverse childhood experiences in Wave 4 and random effects of individual and wave, by using mixed effects logistic regression models

Multiple logistic regressions were used to present the association between the four lifestyle trajectory groups with suicide ideation (**Appendix Table** **3**), alcohol use (**Appendix Table** **4**) and NSSI (**Appendix Table** **5**), while logistic mixed effects model for depressive symptoms (**Appendix Table** **6**), adjusted for time (for depressive only), age, BMI, sex, family income, low maternal education, warm parenting and adverse childhood experiences in Wave 4. “Unhealthy persistent” lifestyle trajectory was associated with an odds ratio (OR) of 2.86 (95%CI, 2.15–3.81) for suicide ideation, 2.16 (95%CI, 1.39–3.35) for depressive symptoms, 2.53 (95%CI, 1.78–3.61) for alcohol use, and 1.35 (95%CI, 1.09–1.67) for NSSI compared with children with healthy lifestyle trajectory (Fig. 3).

In addition, children in “unhealthy mitigation” trajectory also had a higher chance of suicide ideation [OR (95%CI) = 1.86 (1.37–2.52)], and depressive symptoms [OR (95%CI) = 1.96 (1.23–3.12)] compared to the healthy group (Fig. 3).

Warm parenting were significantly associated with lower risk of psychopathological outcomes, while ACEs were significantly associated with higher risk of depressive symptoms (**Appendix Table** **6**), suicide ideation (**Appendix Table** **3**), and NSSI (**Appendix Table** **5**).

## Discussion

Using data from a cohort of children over the transition from childhood to adolescence, this is the first study to our knowledge to map and identify the developmental trajectories of multiple lifestyle factors, as well as its distinct relations with psychopathological outcomes over 5 years of follow-up. Our findings identified that less than 40% of Chinese children belonged to a healthy lifestyle, and 1 in 5 were classified into a persistent unhealthy lifestyle combining stable-high screen time and SSBs intake with abrupt decrease in sleep duration (less than 8 h/d), which was independently associated with two-folds elevated odds for multiple domains of psychopathology, including suicide ideation, depressive symptoms, alcohol use, and non-suicidal self-harm.

The transition from childhood to adolescence has been seen as a crucial phase where unhealthy behaviors are adopted or discarded and tend to cluster as a result of changing roles and situations. In the 4-year longitudinal data of 389 children aged 10 and 14 years, Landsberg et al. identified three distinct lifestyle clusters: ‘low activity and low-risk behaviour’ cluster, ‘high media time and high-risk behaviour’ cluster and ‘high activity and medium-risk behaviour’ cluster [34]. To date, there are increasing investigations of longitudinal trajectories of multiple health behaviors in children and adolescents [35, 36]. However, most of the longitudinal trajectory for variables of interest were analyzed for individual healthy behavior separately rather than jointly, potentially overestimating the effect of single health behaviors as unhealthy behaviors tend to cluster [37] particularly evident during adolescence. The virtue of the group-based multi-trajectory modeling used in this study is that it highlights heterogeneity in the linkage between trajectories of multiple intrinsically relevant behaviors [33].

Evidence is accumulating on the mental health effects of modifiable lifestyle patterns among children and adolescents, however, most of these studies examined the association for specific behaviors. For example, researches showed that healthy lifestyle such as sufficient physical activity and sleep associated with a lower risk of youth suicidal ideation [38]. One recent meta-analysis from Korczak et al. [10] suggested that physical inactivity was associated with increased concurrent depressive symptoms; but the association with future depressive symptoms was weak due to a small number of longitudinal studies. Babic et al. [39] and others [40] found a clear inverse association between total recreational screen-time and computer use with psychological well-being. Results in the present study are in accordance previous findings, indicating the vulnerability for psychopathological outcomes during adolescence is associated with distinct trajectories of unhealthy lifestyle factors.

Our results that adolescents in “unhealthy mitigation group” showed similar risk for subsequent suicide ideation and depressive symptoms with those in “persistent unhealthy group”, which was in line with Carli and his colleagues [36]. In their cross-sectional study of 12,395 adolescents from 11 European countries, the authors identified three groups of pupils, including high-risk group (13.2%) who scored high on all risk behaviors and “invisible risk” group (29%) who were positive for high screen time, sedentary behavior and reduced sleep. Results suggested that these two groups had similar prevalence of suicidal thoughts, subthreshold depression and depression. Children in the “unhealthy mitigation” group are characterized with high-decreasing screen time, low-increasing physical activity and high-decreasing SSBs intake, suggesting developmental vulnerability may psychopathological vulnerability might be initially set by early childhood risky health-related behaviors, improvement in healthy lifestyle during adolescence are not adequate enough to mitigate the effects of existing risks. This population of children may represent an important “invisible” intervention target group for potentially reducing psychopathology and other untoward outcomes in adolescence.

It’s interesting to note that children from the persistent unhealthy group reported stable-moderate level of physical activity, suggesting that greater vulnerability for psychopathology might have been associated with other potential risk factors, e.g. low parenting warmth and high adverse childhood experiences, as presented in this group of children. These findings indicate that parenting quality matters and may lower the risk of psychopathological outcomes and further suicide risk in adolescence for children with persistent unhealthy lifestyle.

The present study comprehensively assessed the associations between a combination of multiple repeatedly-assessed lifestyle behaviors and various psychopathological outcomes in Chinese children and adolescents. This study applied an innovative analytic approach—group-based multi-trajectory analysis—that allows for the identification of diverse developmental trajectories of four lifestyle behaviors.

The contributions of this study should be viewed with its limitations in mind. First, data were collected through parent- or self-report with some single-item measures, which may have limited reliability and potentially cause misclassification. The lack of accelerometry-measured physical activity data limited our ability to capture the complexity of the physical activity and sedentary behavior patterns comprehensively. Accelerometer-derived measures of sleep and rest-activity patterns and use them to further understand the biology of sleep. Besides, the mix of parent-report in the first wave combined with adolescent report in later waves introduces instrumentation bias. Another limitation of this study is the reliance on self-reported of usual sleep hours to reflect an average of sleep durations. Future work should use multiple measures the characteristics of sleep quality, including objective measures. Thirdly shorter sleep length can also be regarded as one of the symptoms of depression (one of outcome variables), this partial correlation between these parameters might yield inflated correlations due to shared method variance. Fourthly, a small proportion of adolescents (10%) missed questions related to psychopathological outcomes despite a similar demographic characteristics and baseline depressive symptoms compared with those included. Furthermore, this study has the inherent limitation of residual confounding due to unmeasured covariates, such as genetic background, which could make associations a poor estimate of causal effects. Finally, this study was conducted in one specific geographic region in China, and the findings may not be generalizable to adolescents across China.

## Conclusion

This prospective cohort of Chinese children provides convincing epidemiological evidence that an unhealthy lifestyle during childhood was associated with more than two-fold elevated risk for suicide ideation, non-suicidal self-harm, depressive symptoms and alcohol use over a period of 5 years. This study provides a basis for quantitative estimates for the potential effect of a population-based lifestyle intervention on the growing burden of mental disorders in China, especially among adolescents and emerging adults. Extended follow-up of this cohort would provide further evidence of the long-term impact of overall lifestyle modification in mental health promotion.

## Supplementary Information

**Additional file 1 Table 1.** Model fit statistics of the group-based multi-trajectory modeling for 2- to 5-class solutions. **Table 2.** Baseline characteristics of participants with missing data. **Table 3.** Multiple logistic regression models for suicide ideation and covariates in different lifestyle trajectory groups. **Table 4.** Multivariate logistic regression models for alcohol use and covariates in different lifestyle trajectory groups. **Table 5.** Multivariate logistic regression models for deliberate self-harm and covariates in different lifestyle trajectory groups. **Table 6.** Multivariate logistic regression models for depressive symptoms and covariates in different lifestyle trajectory groups.

## Data Availability

Data and materials of this study are available from the corresponding author upon reasonable request.

## Abbreviations

PA: Physical activity
SSBs: Sugar-sweetened beverages
