# Supplementary material for: Joint trajectories of life style indicators and their links to psychopathological outcomes in the adolescence
Source: BMC Psychiatry. 2021 Aug 17;21:407. doi: 10.1186/s12888-021-03403-y (PMC8369712; doi:10.1186/s12888-021-03403-y)
Supplement: Supplementary file 1 — Additional file 1 Table 1. Model fit statistics of the group-based multi-trajectory modeling for 2- to 5-class solutions. Table 2. Baseline characteristics of participants with missing data. Table 3. Multiple logistic regression models for suicide ideation and covariates in different lifestyle trajectory groups. Table 4. Multivariate logistic regression models for alcohol use and covariates in different lifestyle trajectory groups. Table 5. Multivariate logistic regression models for deliberate self-harm and covariates in different lifestyle trajectory groups. Table 6. Multivariate logistic regression models for depressive symptoms and covariates in different lifestyle trajectory groups. [file 12888_2021_3403_MOESM1_ESM.docx]

**Appendix Table 1** Model fit statistics of the group-based multi-trajectory modeling for 2- to 5-class solutions

|  | **AIC** | **BIC** |
| --- | --- | --- |
| 2 class | 47851.15 | 47958.68 |
| 3 class | 47596.50 | 47741.25 |
| **4 class** | **47214.42** | **47429.49** |
| 5 class | 47262.35 | 47481.55 |

AIC= Akaike’s information criterion; BIC=sample size-adjusted Bayesian information criterion.

**Appendix Table 2** Baseline characteristics of participants with missing data

| **Groups** | **Depressive symptoms** | | | **Suicide ideation**  (n=213) | **Alcohol use**  (n=214) | **Non-suicidal self-harm**  (n=214) |
| --- | --- | --- | --- | --- | --- | --- |
|  | Wave 2 (n=81) | Wave 3 (n=128) | Wave 4  (n=213) |  |  |  |
| **Boys** |  |  |  |  |  |  |
| Missing | **56 (69.1)** ^†^ | 78 (60.9) | 126 (59.2) | 127 (59.3) | 127 (59.3) | 126 (59.2) |
| Included | 1048 (55.4) | 1026 (55.6) | 978 (55.5) | 977 (55.5) | 977 (55.5) | 978 (55.5) |
| **Age at Wave 1** |  |  |  |  |  |  |
| Missing | **7.8± 0.9 ^§^** | **8.3± 1.0** ^†^ | **8.6± 0.7 ^§^** | **8.6± 0.7 ^§^** | **8.6± 0.7 ^§^** | **8.6± 0.7 ^§^** |
| Included | 8.1± 0.9 | 8.1± 0.9 | 8.1± 0.9 | 8.1± 0.9 | 8.1± 0.9 | 8.1± 0.9 |
| **Body mass index** | |  |  |  |  |  |
| Missing | 16.1± 2.9 | 16.7± 2.7 | **17.5±3.1 ^‡^** | **17.5±3.1 ^‡^** | **17.5±3.1 ^‡^** | **17.5±3.1 ^‡^** |
| Included | 16.8± 2.9 | 16.8± 2.9 | 16.7± 2.8 | 16.7± 2.8 | 16.7± 2.8 | 16.7± 2.8 |
| **Family income <2000 yuan/month** | |  |  |  |  |  |
| Missing | 3 (5.2) | 8 (6.9) | 8 (4.4) | 8 (4.4) | 8 (4.4) | 8 (4.4) |
| Included | 77 (4.6) | 71 (4.3) | 72 (4.6) | 72 (4.6) | 72 (4.6) | 72 (4.6) |
| **Baseline SMFQ** |  |  |  |  |  |  |
| Missing | 3.3±3.0 | 3.3±2.7 | 3.0±2.5 | 3.0±2.5 | 3.0±2.5 | 3.0±2.5 |
| Included | 2.9±2.8 | 2.9±2.8 | 2.9±2.8 | 2.9±2.8 | 2.9±2.8 | 2.9±2.8 |

^a^ Compared with included group, † *P*<0.05; ‡ *P* <0.01; § *P* <0.001

SMFQ= short version of Mood and Feelings Questionnaire

**Appendix Table 3** Multiple logistic regression models for suicide ideation and covariates in different lifestyle trajectory groups

| **Variables** | **Suicide ideation** | | |
| --- | --- | --- | --- |
|  | **Odds ratios** | **95% CI** | ***P* value** |
| **Trajectories of lifestyle facotrs** |  |  |  |
| Healthy | Ref. |  |  |
| Suboptimal healthy | 1.23 | 0.92, 1.65 | 0.491 |
| Unhealthy mitigation | **1.86** | **1.37, 2.57** | **<0.001** |
| Unhealthy persistent | **2.86** | **2.15, 3.81** | **<0.004** |
| **Age** | **1.06** | **1.00, 1.13** | **0.049** |
| **BMI** | 1.01 | 0.98, 1.03 | 0.704 |
| **Female** | **1.29** | **1.04, 1.59** | **0.020** |
| **Low maternal education ^a^** | **1.32** | **1.14, 1.52** | **<0.001** |
| **Household income** | **0.83** | **0.73, 0.95** | **0.008** |
| **ACEs score** | **1.39** | **1.33, 1.45** | **<0.001** |
| **Warm parenting score (0-15)** | **0.91** | **0.88, 0.93** | **<0.001** |

^a^ Lower than high school;

**Appendix Table 4** Multivariate logistic regression models for alcohol use and covariates in different lifestyle trajectory groups

| **Variables** | **Alcohol use** | | |
| --- | --- | --- | --- |
|  | **Odds ratios** | **95% CI** | ***P* value** |
| **Trajectories of lifestyle facotrs** |  |  |  |
| Healthy | Ref. |  |  |
| Suboptimal healthy | **1.56** | **1.10, 2.20** | **0.005** |
| Unhealthy mitigation | 1.20 | 0.79, 1.83 | 0.001 |
| Unhealthy persistent | **2.53** | **1.78, 3.61** | **<0.001** |
| **Age** | 1.03 | 0.95, 1.12 | 0.428 |
| **BMI** | 0.99 | 0.96, 1.02 | 0.689 |
| **Female** | **0.71** | **0.55, 0.92** | **0.009** |
| **Low maternal education ^a^** | 1.10 | 0.80, 1.24 | 0.234 |
| **Household income ^b^** | 1.11 | 0.95, 1.31 | 0.171 |
| **ACEs score** | 1.00 | 0.94, 1.07 | 0.915 |
| **Warm parenting score (0-15)** | **0.94** | **0.91, 0.97** | **<0.001** |

^a^ Lower than high school.

**Appendix Table 5** Multivariate logistic regression models for deliberate self-harm and covariates in different lifestyle trajectory groups

| **Variables** | **Non-suicidal self-harm** | | |
| --- | --- | --- | --- |
|  | **Odds ratios** | **95% CI** | ***P* value** |
| **Trajectories of lifestyle facotrs** |  |  |  |
| Healthy | Ref. |  |  |
| Suboptimal healthy | 1.01 | 0.84, 1.21 | 0.519 |
| Unhealthy mitigation | 0.82 | 0.66, 1.02 | 0.099 |
| Unhealthy persistent | **1.35** | **1.09, 1.67** | **0.002** |
| **Age** | 0.98 | 0.94, 1.02 | 0.284 |
| **BMI** | 1.01 | 0.99, 1.03 | 0.286 |
| **Female** | 0.96 | 0.83, 1.11 | 0.597 |
| **Low maternal education ^a^** | 1.09 | 0.98, 1.20 | 0.112 |
| **Household income** | **0.82** | **0.75, 0.90** | **<0.001** |
| **ACEs score** | **1.41** | **1.30, 1.52** | **<0.001** |
| **Warm parenting score (0-15)** | **0.95** | **0.93, 0.97** | **<0.001** |

^a^ Lower than high school.

**Appendix Table 6** Multivariate logistic regression models for depressive symptoms and covariates in different lifestyle trajectory groups

| **Variables** | **Depressive symptoms** | | |
| --- | --- | --- | --- |
|  | **Odds ratios** | **95% CI** | ***P* value** |
| **Trajectories of lifestyle facotrs** |  |  |  |
| Healthy | Ref. |  |  |
| Suboptimal healthy | 1.41 | 0.56, 1.46 | 0.148 |
| Unhealthy mitigation | **1.96** | **1.23, 3.12** | **0.004** |
| Unhealthy persistent | **2.16** | **1.39, 3.35** | **0.001** |
| **Age** | **1.39** | **1.18, 1.63** | **<0.001** |
| **BMI** | 1.03 | 0.99, 1.07 | 0.122 |
| **Female** | 1.02 | 0.73, 1.42 | 0.914 |
| **Low maternal education ^a^** | 0.99 | 0.80, 1.24 | 0.968 |
| **Household income** | 1.02 | 0.83, 1.24 | 0.894 |
| **ACEs score** | **1.41** | **1.30, 1.52** | **<0.001** |
| **Warm parenting score (0-15)** | **0.88** | **0.85, 0.92** | **<0.001** |

^a^ Lower than high school;

Random intercept, coefficient estimate was 1.50 (95%CI:1.26, 1.79).
